# Changes in surface glycosylation and glycocalyx shedding in *Trichobilharzia regenti* (Schistosomatidae) during the transformation of cercaria to schistosomulum

**DOI:** 10.1371/journal.pone.0173217

**Published:** 2017-03-15

**Authors:** Jana Řimnáčová, Libor Mikeš, Libuše Turjanicová, Jana Bulantová, Petr Horák

**Affiliations:** Department of Parasitology, Faculty of Science, Charles University, Viničná 7, Prague 2, Czech Republic; Universidade Guarulhos, BRAZIL

## Abstract

The invasive larvae (cercariae) of schistosomes penetrate the skin of their definitive hosts. During the invasion, they undergo dramatic ultrastructural and physiological transitions. These changes result in the development of the subsequent stage, schistosomulum, which migrates through host tissues in close contact with host’s immune system. One of the striking changes in the transforming cercariae is the shedding of their thick tegumental glycocalyx, which represents an immunoattractive structure; therefore its removal helps cercariae to avoid immune attack. A set of commercial fluorescently labeled lectin probes, their saccharide inhibitors and monoclonal antibodies against the trisaccharide Lewis-X antigen (Le^X^, CD15) were used to characterize changes in the surface saccharide composition of the neuropathogenic avian schistosome *Trichobilharzia regenti* during the transformation of cercariae to schistosomula, both *in vitro* and *in vivo*. The effect of various lectins on glycocalyx shedding was evaluated microscopically. The involvement of peptidases and their inhibitors on the shedding of glycocalyx was investigated using *T*. *regenti* recombinant cathepsin B2 and a set of peptidase inhibitors. The surface glycocalyx of *T*. *regenti* cercariae was rich in fucose and mannose/glucose residues. After the transformation of cercariae *in vitro* or *in vivo* within their specific duck host, reduction and vanishing of these epitopes was observed, and galactose/N-acetylgalactosamine emerged. The presence of Le^X^ was not observed on the cercariae, but the antigen was gradually expressed from the anterior part of the body in the developing schistosomula. Some lectins which bind to the cercarial surface also induced secretion from the acetabular penetration glands. Seven lectins induced the shedding of glycocalyx by cercariae, among which five bound strongly to cercarial surface; the effect could be blocked by saccharide inhibitors. Mannose-binding protein, part of the lectin pathway of the complement system, also bound to cercariae and schistosomula, but had little effect on glycocalyx shedding. Our study did not confirm the involvement of proteolysis in glycocalyx shedding.

## Introduction

*Trichobilharzia regenti* (Trematoda, Schistosomatidae) is a neuropathogenic avian schistosome that migrates through the CNS to nasal areas of anatid birds. Its infective larvae, aquatic free-swimming cercariae, are well known as the causative agent of cercarial dermatitis in humans [1], a condition regarded as an emerging disease that currently requires attention in many countries all over the world [2,3].

Cercariae emerging from the snail intermediate host actively penetrate the skin of their definitive bird hosts or accidental (dead-end) mammalian hosts, including humans, and transform to schistosomula [4]. This process is accompanied by a detachment of the cercarial tail and emptying of the penetration glands. In schistosomes, cercarial bodies generally undergo extensive ultrastructural rebuilding that is accompanied by changes in biochemical processes and molecular (antigenic) composition of the tegumental glycocalyx. Transforming larvae form a double outer tegumental membrane with protective function, and shed the highly immunogenic glycocalyx which had protected them against the aquatic environment [5,6]. Much information about the structure of glycocalyx is available from human schistosomes, especially *Schistosoma mansoni*. The entire surface of the cercarial stage is covered by a 1–2 μm thick glycocalyx unusually rich in fucose residues [7,8]. Saccharide molecules represented by a heterogenous population of highly fucosylated glycans are bound to lipids and proteins on the membrane of the cercarial tegument by O-glycosidic bonds via sphingosine and serine or threonine, respectively [9–12]. Further detailed structural studies performed with cercariae and schistosomula have led to additional identification of a range of glycans, including smaller O-glycans with unusual core structures and xylosylated N-glycans, which both carry large amounts of Lewis-X antigen (Le^X^, CD15, 3-fucosyl-N-acetyl-lactosamine) also bound in glycolipids. The transformation of cercaria to schistosomulum is accompanied by extensive changes, characterized by a loss of multifucosylated GalNAcβ1-4GlcNAc (LDN) O-glycoproteins, which are replaced by LDN-rich glycosphingolipids [13–16].

Body surface glycosylation of schistosomes has been extensively studied by lectin binding assays, which confirmed the abundance of fucose residues, especially in the cercarial glycocalyx, and revealed differences in lectin binding patterns among particular life stages [17–23,6].

The mechanism of glycocalyx shedding during the penetration of cercariae into the host is known only to a limited extent. Hypotheses based on indirect evidence suggest that peptidases or (phospho)lipases from cercarial penetration glands might be involved [24–26, 6]. Specific secretions from the glands are in charge for lysis of the surface epithelia and underlying tissues during invasion of the host. Gland contents are released in response to a chemical stimulus at the contact with the host’s skin. The stimuli, mostly unsaturated fatty acids such as linoleic and linolenic acids, seem to be quite uniform for the schistosome species studied so far [27,28]. Some artificial compounds, such as lectins [29], praziquantel [30,28], or a calcium ionophore [28], have also been described as inducers of gland emptying *in vitro*.

Penetration gland apparatus of the schistosome species studied so far consists of two compartments formed by unicellular secretory structures that differ in their position within cercarial body, ultrastructure, and chemical composition. Three pairs of postacetabular glands are located posteriorly and two pairs of circumacetabular glands surround the base of the ventral sucker [31,32]. The specific content of the particular types of penetration glands allows for a differential staining by histological dyes: lithium carmine has affinity to the basophilic postacetabular glands, while the circumacetabular glands are targeted by calcium-binding dyes, such as alizarin [31,28]. The content of avian schistosome penetration glands is still poorly understood in comparison to *S*. *mansoni* and *S*. *japonicum*. In these two species of human blood flukes, proteomic methods identified different types of peptidases and lipid-processing enzymes [33–38]. In *T*. *regenti*, a cysteine peptidase of cathepsin B type occurs in the postacetabular glands [39], some sticky mucosubstances have been found in the postacetabular glands of *S*. *mansoni* and *T*. *regenti* [18,28], and high amounts of calcium have been detected in the circumacetabular glands of both of these species [40,41,28].

Our study focused on qualitative changes in surface glycosylation of *T*. *regenti* during the transformation of cercariae to schistosomula both *in vivo* and *in vitro*, and on microscopic observation of the process of glycocalyx shedding by cercariae either in the presence of linoleic acid, or during the action of exogenous lectins, including mannose-binding protein known to activate the lectin pathway of the complement system. We had also tested the involvement of penetration gland products, such as a cysteine peptidase from the glands, as possible triggers of glycocalyx shedding.

## Materials and methods

### Ethics statement

Maintenance and care of experimental animals has been implemented in accordance with European Directive 2010/63/EU, since the Czech Republic is a member state of the EU, and in accordance with the Czech laws (246/1992 and 359/2012) that regulate biomedical research involving animals. Experiments have been performed with the legal consent of the Professional Ethics Committee of the Faculty of Science, Charles University, i.e. the relevant institutional ethics committee for animal research, and of the Research and Development Section of the Ministry of Education, Youth, and Sports of the Czech Republic” (approval no. MSMT-31114/2013-9), which specifically approved this study. The animal facility, its equipment, animal welfare and accompanying services, including the maintenance of experimental animals, have been approved by the Section of Animal Commodities of the Ministry of Agriculture of the Czech Republic (approval no. 13060/2014-MZE-17214). Experimental animals were euthanized by ketamine/xylazine followed by a cervical dislocation or decapitation.

### Parasites, care, and infection of experimental animals

The life cycle of *Trichobilharzia regenti* has been maintained via laboratory-reared *Radix lagotis* snails (intermediate hosts) and the ducklings of *Anas platyrhynchos* f. *dom*. (final hosts). One-day-old American Pekin ducklings of the Cherry Valley strain (*Anas platyrhynchos* f. *domestica*) were supplied by Perena Ltd., Chlumec nad Cidlinou (Czech Rep.). Ducklings were kept in the animal facility in cages under lamps producing radiant heat–during the first week at 28°C, second week at 25°C, third week at 23°C, and thereafter at 18–20°C, at 16:8 h light/dark cycle. They were fed commercial pellets for ducks (Energys Mini, De Heus Plc., Czech Rep.). The infection of ducks by the cercariae of *T*. *regenti* was performed on 7th day of life.

The cercariae emerged from snails illuminated in beakers filled with tap water which had been left to sit out. The larvae were first concentrated in a small volume of water by employing their positive phototaxis, and then transferred into clean tap water. Cercariae were then used to infect ducklings (see above) and adult laboratory mice (*Mus musculus*, strain C57BL/6 or BALB/c, Harlan UK Ltd.). The mice were kept in the animal facility in cages at 20°C and 12:12 h light/dark cycle, and fed commercial pellets for mice and rats (VKS Pohledští Dvořáci Plc., Czech Rep.). Infections were performed via exposed legs (ducks) or the hind legs and tails (mice) in the dark at room temperature (RT) for 1 h. In all experiments, mice and ducks were exposed to 1,000 and 3,000 cercariae, respectively. The health of the experimentally infected animals was monitored twice a day during the whole experiment. Animals were euthanized by humane methods: anesthesia by i.m. injection of ketamine/xylazine followed by cervical dislocation (mice) or decapitation (ducks). No unexpected deaths occurred during the experiments. Euthanasia and dissections of the experimentally infected animals were performed at specific time points: at the end of patency of *T*. *regenti* infection in ducks 21 days post-infection (dpi), and/or at the time intervals after infection when schistosomula reached the desired phase of development in ducks or mice (see the following paragraph). Worms migrated from the dissected skin or spinal cords into 0.1 M phosphate-buffered saline (150 mM NaCl) pH 7.2 (PBS) in a Petri dish. The cercariae of *T*. *regenti* were also used for *in vitro* transformation and cultivation of one- and three-days-old schistosomula according to a previously published protocol [42]. The cercariae or schistosomula were collected and either fixed (4% paraformaldehyde diluted in PBS, 4°C, 1 h) and washed (4x 10 min in cold PBS with 100 mM glycine, 1x 10 min in PBS) or used directly as living larvae.

The lectin binding studies and immunocytochemistry of Le^X^ antigen were evaluated in fresh cercariae, in *in vitro* cultivated schistosomula (1 and 3 days old), and in the *in vivo* transformed (*ex vivo*) schistosomula (1.5 and 3 hours, 1, 3, 5 and 7 dpi from duck skin or spinal cord; only 7 dpi from mouse spinal cord).

### The binding of lectins to cercariae and schistosomula

The fresh cercariae were transferred into 10 mM HEPES buffer pH 7.2 supplemented with 1 mM CaCl_2_, the living schistosomula were placed in the same buffer with 150 mM NaCl. Thirteen fluorescently labeled lectins (Vector Laboratories; HPA from Invitrogen) of various saccharide binding specificity were used at 20 μg/ml final concentration (lectins and their saccharide inhibitors are listed in Table 1). As a control, particular lectins were preincubated for 20 min with their appropriate saccharide inhibitors (Sigma-Aldrich) at 200 mM final concentrations (excluding inhibitors of UEA-I and LTA, whose concentrations were 100 mM) before being added to the larvae. Parasites (at least 20 individuals) were transferred onto a microscopic slide and incubated with lectin or lectin+inhibitor for 20 min. A reaction of mannan-binding lectin (MBL) with the surfaces of cercariae and schistosomula was tested with non-labeled human recombinant MBL (cat no. 2307-MB/CF; R&D Systems) using an adapted protocol previously applied to *S*. *mansoni* [23]. Live or 4% paraformaldehyde-fixed and washed parasites were first incubated for 1 h with 10 μg/ml lectin in HEPES buffer (see above) containing 1% BSA. All steps were carried out at 4°C and in the dark. After repeated washing, 5 μg/ml anti-MBL polyclonal goat IgG (AF2307-SP; R&D Systems) in HEPES-BSA was added for 1 h. Following next wash, the final incubation was performed with 1 μg/ml anti-goat IgG antibody conjugated with AlexaFluor 488 (Sigma-Aldrich). In the controls, MBL was omitted. Lectin binding to the cercariae and schistosomula was visually evaluated under a fluorescence microscope (Olympus BX51).

**Table 1:** Lectins, saccharide inhibitors, and binding preferences of lectins.

Lectin	Abbreviation	Inhibitor	Binding preferences (terminal sequences)
*Lotus tetragonolobus*	LTA	Fuc	Fucα1→2Galβ1→
*Ulex europaeus*	UEA-I	Fuc	Fucα1→2Galβ1→>(GlcNAcβ1→4)_n_>GalNAcα1→3Galβ1→> GlcNAcβ1→6Gal>Galβ1→3GlcNAcβ1→3Galβ1→4Glc
*Lens culinaris*	LCA	MetMan/MetGlc	Manα1→> Glcα1→>> GlcNAc; Fucα1→6GlcNAc
*Canavalia ensiformis*	ConA	MetMan/MetGlc	Manα1→> Glcα1→> GlcNAcα1→
*Pisum sativum*	PSA	MetMan/MetGlc	Manα1→> Glcα1→>> GlcNAc; Fucα1→6GlcNAc
*Solanum tuberosum*	STA	oligo GlcNAc (β1→4)	branched and linear poly-LacNAc, (GlcNAc)_n_
*Triticum vulgaris*	WGA	oligo GlcNAc (β1→4)	(GlcNAcβ1→4)_n_ > GlcNAcβ1 →4>NeuNAc
*Glycine max*	SBA	GalNAc	GalNAcα,β1→ >>Fucα1→2Galβ1 > Gal
*Helix pomatia*	HPA	GalNAc	GalNAcα1→ > GalNAc > GlcNAc > α-Gal
*Ricinus communis*	RCA-I	Gal, Lac	Galβ1→4GlcNAc > β-Gal >α-Gal >GalNAc
*Griffonia simplicifolia*	GSL-I	Gal/GalNAc	α-GalNAc, α-Gal
*Arachis hypogaea*	PNA	Gal	Galβ1→3GalNAc >>Galβ1→4GlcNAc>Gal = Galβ1→3GlcNAc
*Artocarpus integrifolia*	JAC	GalNAc, Mel	polyspecific lectin, Galβ1→3GalNAcα1 →Ser/Thr, GalNAcα1 →Ser/Thr
*Mannose-binding lectin*	MBL	Man/Glc	polyspecific lectin, GlcNAc→Man→ManNAc, Fuc →maltose→Glc→Gal, GalNAc

Abbreviations in Table 1

Fuc–L-fucose, Gal–D-galactose, GalNAc–N-acetyl-D-galactosamine, Glc–D-glucose, GlcNAc–N-acetyl-D-glucosamine, Lac– α-lactose, Man–D-mannose, Mel–melibiose, MetMan– α-D-methylmanopyranoside, MetGlc– α-D-methylglucopyranoside, NeuNAc–N-acetylneuraminic acid, poly-LacNAc–poly-N-acetyllactosamine, Ser–serine, Thr–threonine

Every experiment was repeated three times.

### Immunocytochemistry of Lewis-X antigen

Monoclonal antibodies against the Le^X^ antigen (anti-CD15 mAb) developed in a cooperating laboratory (provided by Prof. Václav Hořejší, Institute of Molecular Genetics of the Academy of Sciences of the Czech Republic, Prague) were employed for detection of the Le^X^ on the surface of the *T*. *regenti* cercariae and schistosomula according to a published protocol [43] with slight variations. Fixed and washed parasites were blocked in 3% BSA in PBS for 1h and then incubated with the primary antibody (mouse anti-CD15 IgM, 10 μg/ml antibody in 3% BSA in PBS) for 2 h. After 4 washes with cold PBS, the larvae were exposed for 1 h to rabbit anti-mouse IgM secondary antibodies conjugated with Alexa Fluor 488 (Invitrogen, 1:200 in 3% BSA in PBS) and washed again 3 times with cold PBS. Finally, they were embedded into Vectashield Mounting Medium H-1000 (Vector Laboratories) and observed under fluorescence (Olympus BX 51) and confocal (Leica TCS SP2 with Acousto-Optical Beam Splitter) microscopes. In control experiments, anti-CD15 mAb was either omitted or replaced by duck or mouse serum from uninfected individuals (1:50 in 3% BSA in PBS).

### The staining of cercarial gland secretions after *in vitro* stimulation by linoleic acid

Cercarial gland secretions were stained in order to visualize their release and distribution during an artificial stimulation of gland secretion. The cercariae were dropped onto microscopic slides and a stimulating reagent (linoleic acid at final concentration 0.1 μg/ml) was added. After 15–30 min incubation, the cercariae attached to the slide by a dint of the released insoluble gland products. A 1% aqueous solution of neutralized alizarine or lithium carmine was then added.

In other experiments, the adhered products with cercariae were incubated for 15 min with lectins that recognize the surface of cercariae (UEA-I, LTA and JAC; see below for the results of the lectin binding assay). First, cercariae were transferred into 10 mM HEPES buffer, pH 7.2, supplemented with 1 mM CaCl_2_, then linoleic acid was pipetted (15 min), and finally, lectin or lectin+inhibitor in PBS was added at 20 μg/ml + 100 mM final concentration. As a control, we have evaluated the effect of lectins, saccharides, alizarine and lithium carmine alone (without linoleic acid) on cercarial behavior and survival. Additionally, we have also tested lectins combined with alizarine or lithium carmine.

The slides were incubated in moist chambers at RT and the behavior of cercariae was observed under a microscope (Olympus BX51) for 30–60 min at 5-min intervals. The number of cercariae on each slide ranged from 10 to 30. All experiments were repeated at least three times.

### Scanning electron microscopy of cercariae penetrating the skin of ducks

The cercariae were concentrated to ca. 1,000 individuals per ml of water in a 15 ml falcon tube. The opening of the tube was covered by skin excised from the leg of a previously euthanized duck. A defined part of skin surface was thus exposed directly to the cercariae to allow penetration. After 30 min in the dark, an appropriate part of the skin was cut, fixed by hot 4% formaldehyde for 2 min, transferred to fresh 4% formaldehyde, and fixed at RT overnight. The skin with the cercariae was then washed 5 times for 10 min in PBS pH 7.4, dehydrated through ascending grades of ethanol and a critical point dryer (Bal-Tec CPD 030), coated with gold in a sputter coater (Bal-Tec ScD 050), and observed under a scanning electron microscope (JEOL 6380 LV).

### The effect of exogenous lectins on the cercariae

When applying fluorescent lectins to larvae, we observed that in some cases, the living cercariae started to release the products of their penetration glands and remove the bound lectins from their surface. The effects of lectins and their saccharide inhibitors on cercariae were therefore studied in separate experiments. Each of the 14 lectins (Table 1) was applied to the cercariae of *T*. *regenti*. The cercariae were transferred into 10 mM HEPES buffer pH 7.2 with 1 mM CaCl_2_. Lectins were used at 20 μg/ml, 10 μg/ml, and 1 μg/ml final concentrations. As controls, lectins were preincubated for 20 min with a 0.1 M solution of the appropriate sugar inhibitor (Table 1). Moreover, 7 lectins chosen based on their obvious effect on glycocalyx shedding (UEA-I, LTA, Con A, LCA, JAC, SBA and MBL) were tested in combination with saccharides which were not expected to bind to their binding sites, i.e., with non-inhibitory sugars. Cercariae were transferred onto microscopic slide, treated either with lectin, saccharide or lectin+saccharide and observed under a fluorescence microscope (Olympus BX51) for 30–60 min at intervals of 5 min. The slides were kept in moist chambers in the dark. The number of cercariae on each slide ranged from 10 to 30. All experiments were repeated at least three times. During these experiments, we evaluated the binding of lectins to cercarial surface and the stimulation/inhibition of release of the gland content and glycocalyx shedding by the cercariae.

### The effect of recombinant cathepsin B2 and peptidase inhibitors on glycocalyx shedding by the cercariae

To test whether glycocalyx shedding by cercariae may be triggered by endogenous proteolytic enzymes, a recombinant form of the cysteine peptidase cathepsin B2 from postacetabular glands of *T*. *regenti* cercariae was produced and purified as previously described [39]. The cercariae (10–30 individuals) were dropped onto microscopic slides and active recombinant cathepsin B2 was added in 50 mM MES buffer pH 6 at a final concentration of 10 μg/ml. As a control, we used cercariae in the same buffer. After 15–30 min incubation, we applied lectins (20 μg/ml) which had been shown in previous experiments to function as markers of cercaria-to-schistosomulum transformation (UEA-I, PNA); observation of the larvae under the fluorescence microscope started immediately after the addition of lectins and proceeded for 30 min.

The next experiment was based on the assumption that if a peptidase from cercarial penetration glands participated in the shedding of glycocalyx, corresponding peptidase inhibitor should block this process. Three groups of peptidase inhibitors were chosen (all from Sigma-Aldrich): leupeptin (100 μM), aprotinin (2 μg/ml) and TLCK (100 μM) as representatives of serine peptidase inhibitors, EGTA and EDTA (both 5 mM) as inhibitors of metallopeptidases and E-64 (10 μM) and E-64d (a membrane-permeable analogue, 10 μM) as cysteine peptidase inhibitors. Cercariae were first incubated with the individual inhibitors (15 min) and then linoleic acid was added at a final concentration of 0.1 μg/ml. Cercariae with inhibitors and without linoleic acid were used as controls. The effect of inhibitors on cercariae, gland emptying, and glycocalyx shedding was evaluated under a microscope after the addition of fluorescent lectins (UEA-I, JAC) or 1% aqueous solution of lithium carmine. All experiments were repeated three times.

## Results

### The binding of lectins to larval surface saccharides

The profile of lectin binding to the surfaces is significantly different between the cercariae and schistosomula of *T*. *regenti*. The cercariae were labeled by lectins with specificity for Fuc (UEA-I, LTA), Man/Glc (Con A, LCA, PSA), GlcNAc (moderate reaction with WGA) and GalNAc/Gal (JAC). With the exception of WGA (schistosomula), RCA-I (cercarial surface) and JAC (schistosomula), all reactions could be blocked by the appropriate saccharide inhibitors. The transformation of cercariae to schistosomula was generally accompanied by a reduction in lectin binding (Table 2). Schistosomula were characterized by a complete disappearance of ligands available for fucose-specific lectins UEA-I and LTA, and a reduction of ligands for mannose/glucose-specific lectins Con A, LCA, and PSA. Moreover, the transformation to schistosomula could be detected by enhanced reactivity of the surface with galactose/N-galactosamine specific lectins SBA, PNA, and HPA. Lectins UEA-I, LTA, and PNA have therefore been chosen for other experiments as the most reliable markers of transformation of cercariae to schistosomula.

**Table 2:** Lectin binding to the surface of *T*. *regenti* cercariae and schistosomula obtained from infected ducks and after *in vitro* transformation.

LECTIN	CERCARIAE	1.5 h (*ex vivo*)	3 h (*ex vivo)*	1 day (*ex vivo*)	1 day (*in vitro*)	3 days (*ex vivo*)	3 days (*in vitro*)	5 days (*ex vivo*)
**UEA-I**^§^	++	+/-	+/-	-	-	-	-	-
**LTA**^§^	++	-	-	-	-	-	-	-
**Con A**	++	(+)	(+)	(+)	-	(+)	(+)	(+)
**LCA**	++	(+)	(+)	(+)	+	(+)	(+)	(+)
**PSA**	++	+	+	(+)	(+)	(+)	(+)	(+)
**STA**	-	-	-	-	-	-	-	+
**WGA**	+	++*	++*	++*	++*	+*	++*	+*
**SBA**	-	+	+	+	+	(+)	+	(+)
**GSL-I**	-	-	-	(+)	-	(+)	-	-
**PNA**^§^	-	++	++	++	+	+	++*	+
**RCA-I**	(+)*	-	+	+	(+)	+	+	+
**JAC**	++	+*	+*	++*	++*	+*	++*	+*
**HPA**	-	+	+	+	+	+	+	-
**MBL**	(+)	0	0	0	0	0	0	+

Evaluation of fluorescence

++ strong reaction, + moderate reaction, (+) weak reaction,—no reaction

* non-specific reaction

^**§**^ lectin marker of transformation, +/- some larvae, depending on the transformation rate, exhibited different reaction, 0 not tested.

**Fig 1:**
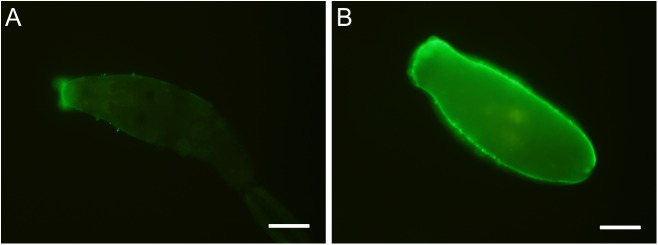
Reaction of human recombinant mannan-binding lectin with the surface of *T*. *regenti* larvae. (A) cercaria. (B) schistosomulum from duck spinal cord 5 days post infection. Prior to immunochemistry, parasites were fixed in 4% paraformaldehyde. Scale bars 50 μm.

The rather polyspecific MBL reacted with the surfaces of both life stages; the reaction was stronger with 5-days-old schistosomula than with the cercariae, and was somewhat more distinct in fixed parasites than in the live stages (Fig 1A and 1B).

### Localization of Lewis-X antigen

**Fig 2:**
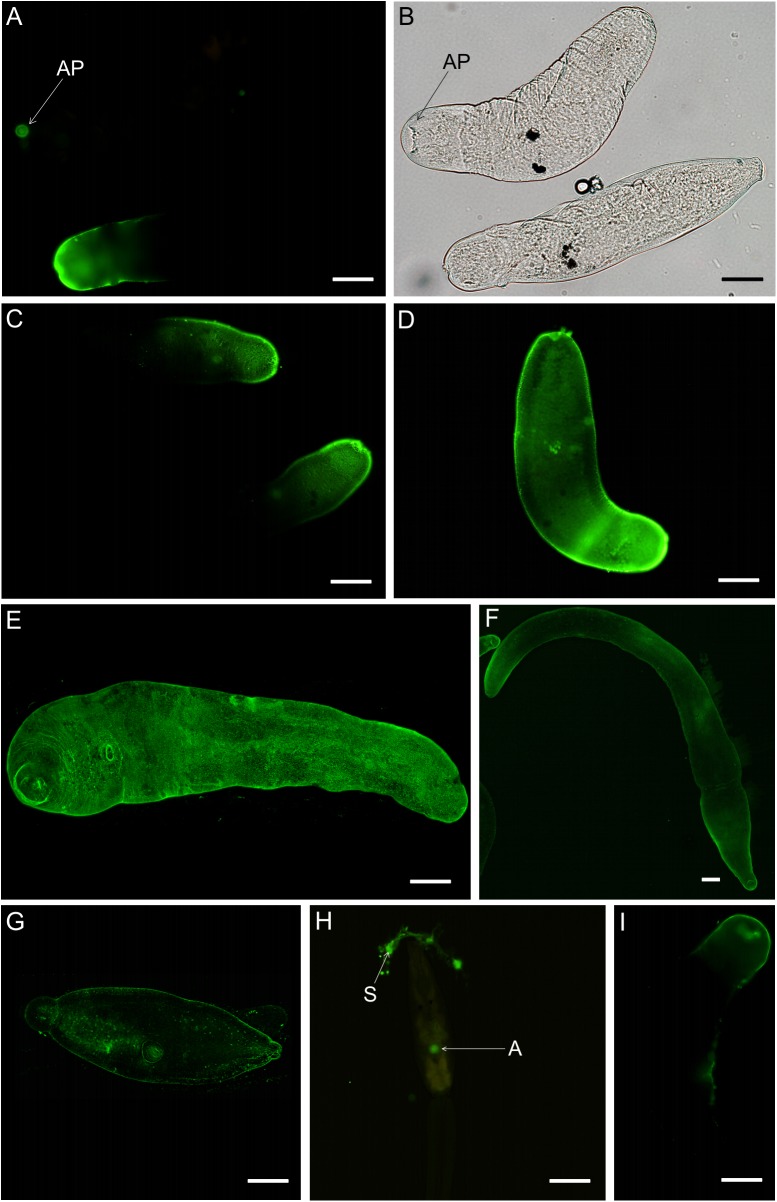
Detection of Lewis X antigen on the surface of *T*. *regenti* larvae by immunocytochemistry with anti-CD15 mAb. (A-F) schistosomula from specific definitive hosts (ducks): (A) 1-day-old schistosomula *ex vivo*, Le^X^ is present only on the anterior surface of one of two larvae of the same age; arrow points to the anterior part of the schistosomulum not reacting with mAb. (B) view of two schistosomula from”A” by light microscopy. (C+D) 3-days-old schistosomula *ex vivo*. (E) 5-days-old schistosomulum *ex vivo*. (F) 7-days-old schistosomulum *ex vivo*. (G) 7-days-old schistosomulum *ex vivo* from a non-specific host (mouse). (H) cercaria–mAb bound only to the secretions of cercarial penetration glands. (I) 3-days-old schistosomulum transformed *in vitro*. AP anterior part of larva, A acetabulum, S secretions of penetration glands. Scale bars 50 μm.

Tegumental surface localization of the Le^X^ antigen was studied using monoclonal anti-CD15 antibodies. Confocal and fluorescence microscopy and specific antibodies detected the Le^X^ neither on the surface of *ex vivo* schistosomula 1.5 h and 3 h old, nor on 1-day-old schistosomula after *in vitro* transformation. Le^X^ epitopes on *T*. *regenti* were first observed at the anterior end of some of 1-day-old schistosomula *ex vivo* (Fig 2A and 2B), and then gradually progressed toward the rear over the entire body of the parasite during its further development. In 3-days-old *ex vivo* schistosomula, in some of the larvae Le^X^ had spread over the entire body, while in others, the antigen was present only over the anterior part of the body (Fig 2C and 2D). In 5- and 7-days-old schistosomula from a specific definitive host (duck), the antigen occupied the entire body surface (Fig 2E and 2F). Seven-days-old schistosomula of *T*. *regenti* from a non-specific host (mouse) also had Le^X^ over their entire body surface (Fig 2G). As for the cercariae, the antibodies bound only to the secretions of their penetration glands, but not to the body surface (Fig 2H). In schistosomula transformed *in vitro* without the presence of any host components, detectable Le^X^ epitopes could be observed on the surface of the anterior part of the body on day 3 after transformation (Fig 2I).

### Staining of cercarial gland secretions and the shedding of glycocalyx

**Fig 3:**
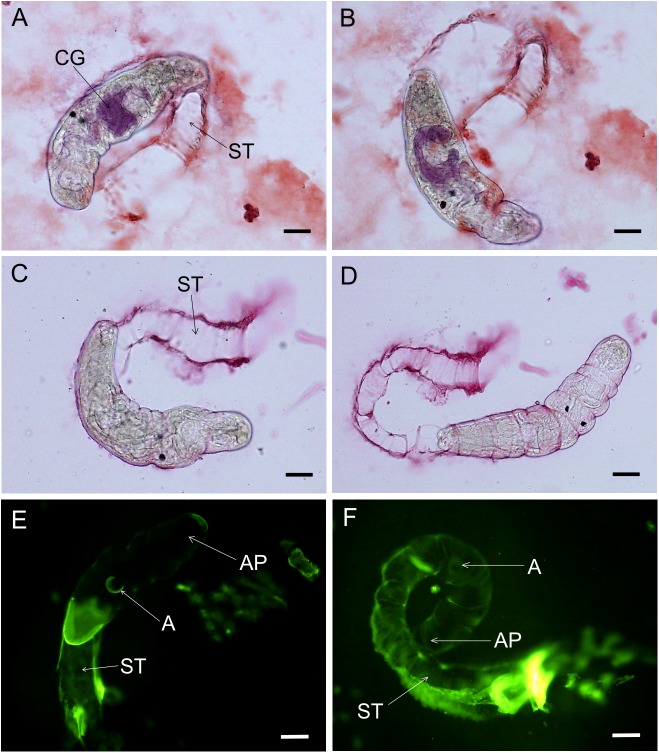
Formation of a “shedding tunnel” from secretions of the penetration glands and glycocalyx in *T*. *regenti* cercariae after stimulation with linoleic acid. (A+B) circumacetabular gland content and secretions lining the shedding tunnel, stained by alizarine. (C+D) lithium carmine-stained postacetabular gland secretions expelled from the glands and lining the shedding tunnel. (E+F) shedding tunnel labeled by fluorescent LTA and loss of lectin ligands on cercarial surface. CG circumacetabular glands, ST shedding tunnel, A acetabulum, AP anterior part of cercaria. Scale bars 50 μm.

During the induction of emptying of penetration glands with linoleic acid and staining of the gland content, we observed strong binding of both post- and circumacetabular secretions to the surface of *T*. *regenti* cercariae. The cercariae were able to shed the material from their surface within a short time (2–15 min). They crawled through the sticky products forming a “shedding tunnel” composed of gland secretions and possibly also the shed glycocalyx components (Fig 3A–3D and S1 Movie).

To prove that glycocalyx is removed from the cercarial surface during the formation of the shedding tunnel after stimulation with linoleic acid, lectins previously confirmed as markers of transformation of the cercariae to schistosomula (UEA-I, LTA and PNA) were applied. After contact with the gland content within the tunnel, cercarial surface lost the ligands for fucose-specific lectins. On the other hand, a reaction with PNA could be observed. The shedding of glycocalyx monitored by labeled lectins started in the anterior part of cercarial body (Fig 3E and 3F).

**Fig 4:**
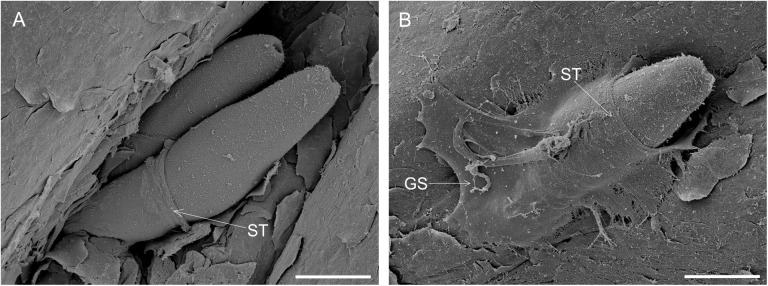
Adhesion of penetration gland secretions to the surface of cercariae penetrating duck skin. Scanning electron micrographs. (A) two cercariae penetrating the skin at the same site. (B) cercaria attached to the skin by gland secretions. ST shedding tunnel around the surface of cercaria, GS acetabular gland secretions adhered to host skin. Scale bars 50 μm.

Scanning electron microscopy of cercariae penetrating the skin of ducks *ex vivo* confirmed that formation of a tunnel composed of acetabular gland secretions is a natural process. Secretions expelled from the penetration glands upon contact of the cercaria with host’s skin cover the front part of the cercarial body and gradually roll toward the rear as the cercaria proceeds towards deeper layers of the skin (Fig 4A). Cercaria is in a close contact with the gland secretions, which also help attach its body to the skin (Fig 4B).

### The effect of exogenous lectins on the cercariae

**Fig 5:**
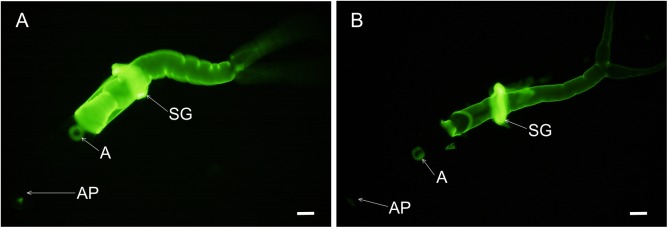
Glycocalyx shedding by *T*. *regenti* cercaria after stimulation with JAC lectin. (A) anterior half of cercarial body shed the glycocalyx. (B) a ring of glycocalyx with bound lectin was shed from the anterior part of body and rolled over the posterior part and the tail stem. ST shed glycocalyx, AP anterior part of cercaria/schistosomulum body, A acetabulum. Scale bars 50 μm.

During a characterization of cercarial surface glycosylation, it has been observed that all of the 14 lectins (without any stimulating reagent) to some extent induce secretion from penetration glands at three different concentrations used (20 μg/ml, 10 μg/ml and 1 μg/ml–with the exception of STA and HPA, which had no effect at 1 μg/ml final concentration). With decreasing lectin concentration, a lower intensity of secretion was observed. With the exception of STA and HPA at the lowest concentration, all of these fluorescently labeled lectins bound to the secretions. Glycocalyx shedding was induced by 7 lectins (UEA-I, LTA, Con A, LCA, SBA, JAC and MBL) at a concentration of 20 μg/ml (Table 3). Lectins with specificity to fucose (UEA-I and LTA) and also JAC were the most effective inducers (Fig 5). The other four lectins had only a limited effect on shedding. It turned out that 5 of these 7 inducing lectins were those which bound strongly to the surface of cercariae; in comparison with these, the binding of MBL was rather weak, while SBA did not react at all. Upon a tight contact with the released gland products, cercarial surface started to be shed and gradually, from the anterior part toward the rear, lost reactivity with the lectins. Differences in the intensity of gland emptying and glycocalyx shedding after incubation with lectins are summarized in Table 3.

**Table 3:** Lectin stimulation of penetration gland emptying and the shedding of glycocalyx in *T*. *regenti* cercariae.

LECTIN	BINDING TO CERCARIAL SURFACE	BINDING TO SECRETIONS	INDUCTION OF GLAND SECRETION	GLYCOCALYX SHEDDING
**UEA-I**	++	++	++++	YES ++
**LTA**	++	++	++++	YES ++
**Con A**	++	++	+++	YES +
**LCA**	++	++	++++	YES +
**PSA**	++	++	++++	NO
**STA**	-	++	++	NO
**WGA**	+	++	+++	NO
**SBA**	-	++	++++	YES +
**GSL-I**	-	++	++++	NO
**PNA**	-	++	++++	NO
**RCA-I**	(+)*	++	+++	NO
**JAC**	++	++	+++	YES ++
**HPA**	-	++	++	NO
**MBL**	0	0	++	YES +

Evaluation of fluorescence

++ strong reaction, + moderate reaction, (+) weak reaction,—no reaction

* non-specific reaction, 0 not tested. The final concentration of lectins was 20 μg/ml.

Evaluation of the intensity of secretion from penetration glands, and glycocalyx shedding.

++++ very strong intensity, +++ strong intensity, ++ moderate intensity, + weak intensity. The intensity takes into account the speed of these processes and the proportion of cercariae which entered this phase on each slide.

To investigate whether the binding sites of lectins (rather than another part of the proteins) participate in the stimulation of glycocalyx shedding, we have preincubated the seven inducing lectins with their saccharide inhibitors. This treatment resulted in inhibiting both lectin binding to cercarial surface and the glycocalyx shedding. Application of saccharides alone did not stimulate any changes in cercariae. Moreover, experiments using combinations of lectins with non-target saccharides (i.e., the application of lectin without any binding preference to the particular saccharide; for compatible and non-compatible combinations see Table 4) showed that glycocalyx shedding took place only when binding of a particular lectin to cercarial surface was not inhibited.

**Table 4:** Effects of lectin/saccharide combinations on cercariae of *T*. *regenti*

LECTIN	SACCHARIDE	BINDING TO SURFACE	GLYCOCALYX SHEDDING
**UEA-I**	**Fuc**	-	N
**UEA-I**	**MetMan/MetGlc**	(+)	50%
**UEA-I**	**Gal/GalNAc**	++	Y
**LTA**	**Fuc**	-	N
**LTA**	**MetMan/MetGlc**	-	20%
**LTA**	**Gal/GalNAc**	-	25%
**ConA**	**MetMan/MetGlc**	-	N
**ConA**	**Fuc**	+	50%
**ConA**	**Gal/GalNAc**	+	50%
**LCA**	**MetMan/MetGlc**	-	N
**LCA**	**Fuc**	(+)	20%
**LCA**	**Gal/GalNAc**	-	N
**SBA**	**Gal/GalNAc**	-	N
**SBA**	**Fuc**	-	33%
**SBA**	**MetMan/MetGlc**	-	33%
**JAC**	**Gal/GalNAc**	-	N
**JAC**	**Fuc**	++	60%
**JAC**	**MetMan/MetGlc**	++	N
**MBL**	**MetMan/MetGlc**	0	10%
**MBL**	**Fuc**	0	20%
**MBL**	**Gal/GalNAc**	0	20%

Evaluation of fluorescence.

++ strong reaction, + moderate reaction, (+) weak reaction,—no reaction, 0 not tested, Y shedding recorded, N shedding absent, % proportion of cercariae undergoing the process.

### The effect of cathepsin B2 and peptidase inhibitors on glycocalyx shedding in *T*. *regenti* cercariae

As demonstrated by a subsequent incubation of cercariae with UEA-I and PNA lectins serving as markers of transformation (see above), incubation of cercariae with a recombinant cathepsin TrCB2 had no effect on glycocalyx shedding.

The treatment of cercariae with particular peptidase inhibitors prior to and during incubation with linoleic acid had no effect on either the secretion from acetabular glands, or release of the tail, or glycocalyx shedding. Inhibitors alone at the concentrations used had no obvious effect on cercarial behavior.

## Discussion

Using lectin binding assays, we have characterized changes in surface saccharide epitopes during *in vivo* and *in vitro* transformation of *T*. *regenti* cercariae to schistosomula, and determined the lectin markers of transformation. The most obvious change was a rapid and massive reduction of ligands of Fuc-specific lectins already in 1.5 h old schistosomula obtained from infected ducks, followed by a complete loss of these ligands in 1-day-old *in vivo* and *in vitro* transformed schistosomula. At the same time, ligands recognized by the Gal-specific lectin PNA appeared on the schistosomula. These results are in accordance with data produced by lectin binding studies on living *T*. *regenti* cercariae [22] and with histological sections of both of the life stages [21,42]. Moreover, comparison with other schistosomes and non-schistosome trematodes had conclusively shown that extensive fucosylation of the cercarial glycocalyx is unique, and represents a general attribute of all the schistosome species studied so far [44,45,9,22]. In the cercarial glycocalyx of *Schistosoma mansoni*, Fuc represents more than 50% of the overall saccharide content; other saccharides include Gal/GalNAc, Glc/GlcNAc, Man and xylose [8,11,14]; sialic acid was not confirmed [9]. Moreover, a reduction and gradual disappearance of lectin-recognized Fuc epitopes, and the emergence of epitopes recognized by some Gal/GalNAc-specific lectins (e.g., PNA) was described in the schistosomula of *S*. *mansoni* and *Trichobilharzia szidati* [46,6]. The swap of saccharide epitopes demonstrably begins in the anterior part of the transforming larvae (where the openings of acetabular glands are located). Human recombinant mannose-binding lectin bound specifically to the surfaces of both the cercariae and the schistosomula. This reaction was stronger in the fixed larvae, which might be an effect of crosslinking of the surface components accompanied by structural changes in the glycocalyx that results in exposing the underlying glycans to the lectin, a phenomenon observed when certain anti-glycan antibodies are used [16].

We noticed some discrepancies between the previously published results and our current data. For instance, variances in the intensities of lectin binding can be explained by the treatment of samples (e.g., fixed vs. live larvae) or by the use of different batches of commercial lectin probes which moreover may be diversely labeled by fluorochromes. The latter possibility makes the relative quantification and comparison of lectin binding assays questionable, because the intensity of fluorescence on lectin-labeled surfaces reflects not only the amount of bound lectin molecules (which is related to the amount of saccharide epitopes), but also the number of fluorochrome molecules on each of the particular lectins (i.e., the fluorochrome to protein molar ratio). In this study, visual evaluation was therefore performed always by the same person using a narrow scale and identical setup of the microscope.

Although the lectin-binding assays demonstrated a general disappearance of fucosylated ligands from the surface of cercariae during their transformation to schistosomula, a gradual increase in the occurrence of Le^X^ trisaccharide {Galβ1→4(Fucα1→3) GlcNAcβ1→} containing fucose was observed in some of the 1-day-old schistosomula using specific antibodies. These epitopes were obviously not detectable by the Fuc-specific lectins used in this study. Again, Le^X^ started to appear on the anterior part of the body, and only in some of the 3-days-old *ex vivo* schistosomula it spread over the entire body surface; it was recorded earlier on larvae transformed *in vivo* than on those from *in vitro* conditions. Reports regarding the presence of Le^X^ in other schistosome species are sometimes contradictory. It has been found on the surface of human schistosomes *S*. *mansoni*, *S*. *japonicum* and *S*. *haematobium* [47]. Its expression is developmentally regulated in *S*. *mansoni*. Immunochemistry confirmed the presence of the antigen on both the schistosomula and the adults, but it seemed absent on the cercariae [48]. In another study, it was found on the surface of cercariae, and on 3 h old, 48 h old, and lung-stage schistosomula, as well as in adults and in eggs [43]. Recent results of mass spectrometry-based glycomics revealed abundant N- and O-linked glycans containing Le^X^ in cercariae, which rapidly declined during their transformation to schistosomula and in the course of further maturation of the worms. In cercarial glycolipids, single Le^X^ terminal motifs, as well as Le^X^ tandem-repeats have been found; they occurred in schistosomula aged up to two weeks, but not in adult worms [15]. Combined glycan microarray and immunofluorescence analyses using a set of characterized anti-glycan monoclonal antibodies showed superficial localization of Le^X^ only in the post-transformational stage of *S*. *mansoni*. It thus appears that Le^X^ motifs present in both N- and O- glycans of cercariae originate from their gland secretions [16]. This may explain the reaction of our antibodies only with the apex of the head organ of *T*. *regenti* cercariae, where the openings of the penetration gland are located [32]. On the other hand, the Le^X^ epitopes might be present in the surface glycolipids of both stages but masked by abundant O-glycans in cercarial glycocalyx, which would make Le^X^ inaccessible for detection by antibodies [49,15,16].

In *S*. *mansoni*, the glycans with Le^X^ antigen can interact directly with the host immune system [43]. In humans, Le^X^ is present on the surface of various cells (mostly in its sialylated form), where it has various important functions in cell signaling and recognition, and serves as a ligand for some lectin-like adhesive proteins [50]. Its expression has been confirmed also in birds [51,52]. Le^X^ is thus an epitope shared by schistosomes and their hosts. Hypotheses and various kinds of evidence for its possible involvement in the parasite’s molecular mimicry and modulation of host immune response appear in several studies [12, 53–55]. Faster changes in terms of detectable Le^X^ epitopes have been observed during *in vivo* rather than *in vitro* transformation. Retarded development after *in vitro* transformation was also documented in the schistosomula of *S*. *mansoni* and *T*. *szidati* [56,6].

In our study, a strong binding of linoleic acid-stimulated secretions to the surface of *T*. *regenti* cercariae was evident. Although a differential emptying of postacetabular and circumacetabular glands in schistosomes has been described by some authors [57], in the present study, both types of glands released their content simultaneously. We are aware, however, that the system might work differently *in vitro* and *in vivo*. Stimulated cercariae formed a “shedding tunnel” and it seemed that they were able to shed the secretions bound to their surface. Similarly, gland products were present along the surface of penetrating larvae of *S*. *mansoni* and *T*. *szidati* [26,6]. Scanning electron microscopy of *T*. *regenti* cercariae penetrating the duck skin provided evidence that formation of the tunnel is not an artefact of the *in vitro* experiment. The binding of fluorescent lectin markers of transformation dramatically differed before and after the contact of the cercarial surface with secretions of the tunnel: Fuc-specific lectins LTA and UEA-I no longer recognized the surface, and there appeared reactivity with PNA. This implies that the glycocalyx has been shed. These changes in glycosylation correspond well with the transformation of the cercariae to schistosomula in *S*. *mansoni* [15,16].

All lectins used in our study induced a discharge of *T*. *regenti* cercarial penetration gland content. This was previously observed in *S*. *mansoni* cercariae exposed to certain lectins, such as PSA and WGA, and the effect was blocked by a pretreatment with inhibiting glycans [29]. Decreasing lectin concentration led to a lower secretion, and preincubation of lectins with specific saccharide inhibitors had no effect on the secretion. Either the binding sites of lectins were not involved in the induction, or the employment of monosaccharides as inhibitors may be insufficient, since lectin binding to saccharides is rather complex [58].

Cercarial surface with secretions is sloughed off in a sleeve-like manner, forming a shedding ring which rolls from the anterior toward the rear part of cercarial body. The bare part of the body then loses the ability to bind lectins which recognize the surface of intact cercariae. This is the process we viewed as the shedding of the glycocalyx. It was markedly induced by some of the lectins strongly binding to cercariae, especially by those with specificity to fucose (UEA-I and LTA) and the polyspecific lectin JAC. Several other lectins, namely SBA, Con A, LCA, and MBL, induced this process with lower efficacy. Interestingly, within the latter group of weak inducers, Con A and LCA bound strongly to cercarial surface, whereas SBA did not bind at all. PSA, on the other hand, while binding strongly to cercarial bodies, did not induce any shedding. This fact opens a question of some yet unclear specificity of the process, which does not seem to consist of just bare crosslinking of any surface glycan structures by a lectin. Preincubation of particular lectins with their inhibiting monosaccharides prevented glycocalyx shedding in the cercariae. The testing of different combinations of inducing lectins with both specific and “incompatible” monosaccharides in some cases resulted in only a partial inhibition of glycocalyx shedding, thus confirming a complex nature of lectin/glycan interactions. Since all lectins at least to some extent stimulated secretion from the penetration glands, it was impossible to observe the shedding of glycocalyx independently of the secretion. However, since some lectins which trigger gland emptying did not induce shedding, it seems that at least two self-contained processes based on specific interactions between the cercarial saccharide ligands and exogenous lectins might be involved.

In order to focus on the role of lectins in cercarial transformation to schistosomula inside the host, we applied recombinant MBL, which is an initiator of lectin pathway of the complement cascade. We reasoned that avoiding complement attack by shedding MBL bound to the surface would be beneficial to the cercariae/schistosomula. It has been previously demonstrated by fluorescent and scanning electron microscopy that some surface carbohydrates of *S*. *mansoni* cercariae and adult worms are ligands for MBL [23]. We confirmed that both cercariae and schistosomula of *T*. *regenti* bind MBL to their surfaces. Functional study with unlabeled MBL and a set of monosaccharides had shown that the lectin is a poor inducer of glycocalyx shedding, and inhibition assays were inconclusive, possibly due to the lectin’s broad specificity. Nevertheless, the effect of other lectins originating from the host (such as ficolins, or collectins and other C-type lectins) on the shedding of schistosome glycocalyx cannot be excluded and it is an issue which should be addressed by future studies. Because these lectins play important roles in innate immunity, their recognition of cercarial structures may be an inseparable part of host-parasite interactions which lead to, e.g., complement activation, opsonization, and cell adherence. They need not always represent a signal for cercarial transformation, although this was clearly demonstrated in our study with exogenous lectins, and a similar effect was observed in some other larval trematode transformations (e.g., *Fasciola hepatica* miracidia to mother sporocysts) [59].

To verify a previous asumption that peptidases from the penetration glands of *S*. *mansoni* cercariae may play a role in the shedding of cercarial glycocalyx [24,26], we tested the role of the recombinant cathepsin B2 (the main penetration enzyme contained in the postacetabular glands of *T*. *regenti* cercariae) [39] and selected peptidase inhibitors in glycocalyx shedding. Proteolytic shaving of the surface epitopes by endogenous peptidases is well known from parasitic protists. For instance, in *Entamoeba histolytica*, there are intramembrane rhomboid serine peptidases involved in immune evasion [60]. Up-regulated expression of a rhomboid-like peptidase of a yet unknown function was demonstrated in cercarial germ balls of *S*. *mansoni* [61]. Another example of such peptidases are sheddases, such as the subtilisin-like peptidase of *Plasmodium falciparum* merozoites which originates in micronemes and is further distributed across the surface [62]. To our best knowledge, however, none of these peptidase types has been found in the soluble or tegumental proteomes of schistosome cercariae. Schistosomes, on the other hand, possess several peptidases of metallo-, cysteine and serine classes, many of which are also present in the excretory/secretory products of nematodes, where they contribute to the cuticle molting/exsheathment process [63].

Tests with a defined set of peptidase inhibitors indicated that neither cathepsin B2, nor other peptidases possibly occurring in the gland secretions have any effect on the process of glycoprotein shedding from the surface of *T*. *regenti* cercariae. This is in accordance with the reported resistance of *S*. *mansoni* cercarial glycocalyx to hydrolysis by several peptidases [9]. Moreover, it has been shown that glycoproteins and glycolipids from the cercarial surface of *S*. *mansoni* are released intact, and they are probably shed together with the surface membrane by a proteolysis-independent mechanism [64].

## Conclusions

We have established that the glycosylation of *T*. *regenti* cercarial glycocalyx is similar to other schistosomes in terms of abundance of fucose epitopes. Cercariae react to stimulation with linoleic acid by releasing secretions from both types of their penetration glands. The expelled gland products adhere to the surface of cercariae and are shed by the moving larvae in a sleeve-like manner, forming a “shedding tunnel” that is left behind. The contact of cercarial surface with gland secretions within the tunnel results in a loss of reactivity with fucose-specific lectins and the emergence of galactose/N-acetylgalactosamine epitopes. These changes are seen as markers of glycocalyx shedding. The process starts in the apical part of the transforming larvae, where openings of penetration glands are located. The pattern of lectin binding to larvae transformed in this way *in vitro* resembles the pattern found in larvae transformed *in vivo* within their specific duck hosts. Some of the lectins, mainly (but not exclusively) those which bind strongly to cercarial surface, can trigger secretion from the penetration glands and the shedding of glycocalyx. We believe this could be part of the parasite’s immunoevasive strategies. On the other hand, the mannose-binding lectin which is involved in the complement cascade within the hosts had but little effect on the cercariae. Even so, the effect of exogenous lectin binding on the transformation of the cercarial surface was obvious and will be further studied. In transforming larvae, Lewis-X antigen motifs recognizable by monoclonal antibodies gradually spread over the surface and this process is completed within ca. 3 days *in vivo*. We did, however, find a high level of diversity among the individual larvae in terms of the speed of surface glycosylation changes, a fact which probably reflects physiological heterogeneity within the parasite population. And finally, our results did not support some previously formulated hypotheses regarding the involvement of penetration gland peptidases in the process of glycocalyx shedding in schistosome cercariae.

## Supporting information

S1 Movie Time-lapse photography of the formation of the shedding tunnel by cercaria of *T*. *regenti*. Secretion from the penetration glands was stimulated by addition of linoleic acid and visualized by staining with lithium carmine. Frames were taken in the intervals of 30 s.
(WMV)
